# Appendectomy, cholecystectomy and diagnostic laparoscopy conducted before pregnancy and risk of adverse birth outcomes: a nationwide registry-based prevalence study 1996–2015

**DOI:** 10.1186/s12884-020-2796-3

**Published:** 2020-02-13

**Authors:** Anne Staub Rasmussen, Christian Fynbo Christiansen, Niels Uldbjerg, Mette Nørgaard

**Affiliations:** 1 Department of Clinical Epidemiology, Aarhus University Hospital, Olof Palmes Allé 43-45, 8200 Aarhus N, Aarhus, Denmark; 2 Aarhus University, Aarhus, Denmark; 3 Department of Obstetrics and Gynecology, Aarhus University Hospital, Aarhus, Denmark

**Keywords:** Pregnancy, Surgical procedures operative, Prevalence, Epidemiology, Appendectomy, Denmark, Cholecystectomy, Laparoscopy

## Abstract

**Background:**

Non-obstetric surgery conducted during pregnancy may increase the risk of adverse birth outcomes like small for gestational age, preterm birth, and miscarriage. Mechanisms are unclear but possibly longer lasting. We examined whether appendectomy, cholecystectomy and diagnostic laparoscopy conducted before pregnancy affect these outcomes.

**Methods:**

This nationwide Danish prevalence study included all pregnancies during 1996–2015 that had an appendectomy, cholecystectomy or diagnostic laparoscopy registered before last menstrual period in the years 1992–2015. We excluded pregnancies with surgery and categorized pre-pregnancy surgery according to timing (0–11, 12–23, and 24+ months before last menstrual period). Outcomes were small for gestational age, late preterm birth (32–37 weeks), early preterm birth (22–31 weeks) and miscarriage (7–21 weeks). We computed absolute risks and used logistic regression comparing pregnancies with surgery 0–11 or 12–23 to 24+ months before last menstrual period, computing odds ratios for each outcome, adjusting for maternal age and smoking.

**Results:**

We identified 15,939 pregnancies with appendectomy, 12,869 pregnancies with cholecystectomy and 19,330 pregnancies with diagnostic laparoscopy. The absolute risk of small for gestational age was 2.2% for patients with appendectomy 0–11 months before last menstrual period, 3.2% 12–23 months before compared with 2.2% when appendectomy was conducted more than 24 months before (adjusted OR 0.95 (95% CI; 0.65 to 1.31) and 1.37(95% CI;1.00 to 1.86). For early preterm birth, the absolute risks were 0.7, 0.5 and 0.8%, for late preterm birth 4.8, 4.4 and 4.7% and for miscarriage 5.7, 6.2 and 5.4%.We observed similar results for cholecystectomy. For diagnostic laparoscopy 0–11 months before pregnancy we found increased risks of small for gestational age (4.0, 2.8 and 2.6%) and late preterm birth (5.9, 5.0 and 4.8%).

**Conclusions:**

We found no increased risk of adverse birth outcomes among pregnancies with appendectomy or cholecystectomy conducted within 2 years before pregnancy compared to more than 2 years before pregnancy. The increased risks 0–11 months after diagnostic laparoscopy are likely explained by confounding by underlying indication. It appears safe to become pregnant any time following appendectomy and cholecystectomy, but, probably depending on indication, attention should be payed 0–11 months after diagnostic laparoscopy.

## Background

Appendectomy, cholecystectomy, diagnostic laparoscopy and other non-obstetric surgery conducted during pregnancy may affect the risk of adverse obstetrical outcomes, including increased risk of preterm birth [1–4], small for gestational age (SGA) and low birth weight [1, 4]. Potential pathways include not only a direct physical effect of surgery or the underlying condition indicating the surgery, but also microbiome alterations (especially oral and intestinal) [5] and increased pro-inflammatory mediator levels [6]. Microbiome alterations may persist for months and could thus potentially affect a pregnancy starting after surgery [7]. A large cohort study found that infants born after maternal bariatric surgery had lower gestational age and increased risk of being small for gestational age than infants born in a matched cohort of women without bariatric surgery [8]. These findings could have various explanations, eg. malabsorption, surgical complications and microbiome changes. Few, if any, studies have examined whether appendectomy, cholecystectomy, or diagnostic laparoscopy before pregnancy has an impact on birth outcomes. Our objective was therefore to examine whether these procedures conducted up to 2 years before pregnancy were associated with increased risk of adverse birth outcomes.

## Methods

This national prevalence study was based on a population of 5.7 million inhabitants in Denmark [9], with an average of 1.3 million female inhabitants aged 15–54 years each year in the years 1996–2015 [9]. All Danish citizens have free and equal access to hospital and specialist treatment through our tax-supported healthcare system [10]. Individual-level linkage of all Danish population-based registries was allowed for through the Civil Personal Registration-number assigned at birth or immigration [11]. This number contains information on birth date and sex. We used the Danish National Patient Registry (DNPR) [12] and the Danish Medical Birth Registry (DMBR) [13] to obtain information on appendectomy, cholecystectomy and diagnostic laparoscopy before pregnancy, surgical procedures during pregnancy, fetal outcomes and relevant covariates.

Through DMBR and DNPR, we identified all Danish female citizens aged 15–54 years who delivered during the period 1995–2016. We restricted to singleton births, because multiple births are associated with both lower fetal weight [14] and lower gestational age at birth [14]. Diagnosis codes are assigned to each patient at day of discharge from hospital or outpatient clinic and registration in the DNPR is mandatory for all Danish hospitals. Non-psychiatric hospital admissions have been recorded since 1977 and, emergency room contacts and contacts to hospital specialist clinics have been registered since 1995. Diagnoses are coded according to the International Classification of Diseases, 8th edition (ICD-8) until 1994 and the 10th edition (ICD-10) thereafter [15]. Surgical procedure codes are registered after surgery according to the Danish version of Nordic Medico-Statistical Committee Classification of Surgical Procedures [16] from 1996. From 1971 to 1995, they were registered according to the Danish Classification of Surgical Procedures and Treatments.

We extracted information on birth weight and gestational age from the DMBR, which was established in 1973. It contains information on all home and hospital deliveries in Denmark. Livebirths regardless of gestational age and stillbirths > 22 weeks are included [17]. Data in the registry are collected prospectively by the midwife attending birth, with information on mother and child collected in one record. Available information on newborns include birth date, gender, birth weight, length at birth, fetal presentation, gestational age, multiple pregnancy, Apgar scores, birth presentation, and mode of birth. Maternal information include: number of previous births, parity, age, marital status, smoking status, pre-pregnancy body-mass index and citizenship [15]. We calculated the estimated first day of last menstrual period (LMP) as day of birth or abortion minus gestational age in days at birth or abortion. The LMP was used for calculation of gestational age at time of surgery.

We then restricted the study population to women with appendectomy, cholecystectomy or diagnostic laparoscopy (see Additional file 2) as the latest surgical procedure before LMP in the years 1992–2015 and no major surgical procedures from date of LMP through pregnancy termination. Pregnancies with minor surgical procedures (eg skin procedures and all transluminal endoscopies) and with cesarean section were not excluded (see codes in Additional file 1). We excluded pregnancies with registration of birth within 139 days of the last birth in the same woman, pregnancies with LMP starting before birth in the last pregnancy in the same woman and births with birthweight above 6500 g or below 500 g.

We analyzed appendectomy, cholecystectomy and diagnostic laparoscopy separately. From each of the specific groups, we excluded pregnancies with any major surgical procedure not being appendectomy, cholecystectomy or diagnostic laparoscopy within 23 months before LMP, respectively (see Additional file 1). We computed time between date of surgery and date of LMP and divided it into 0–11, 12–23 and 24+ months before LMP. To assess potential difference in risk of the outcomes over calendar time due to changing guidelines and the technical development in surgery [18], we categorized calendar into year-groups (1996–1999, 2000–2003, 2004–2007, 2008–2011 and 2012–2015).

The outcomes of interest in our study were SGA, early preterm birth, late preterm birth and miscarriage occurring after gestational week 7.

SGA was defined as births with a birth weight more than 2SD below an age- and sex-specific reference (19). We excluded pregnancies with gestational age (GA) < 22 weeks or missing information on birthweight (0.7%), when calculating the risk of SGA. Early preterm birth was defined as births with a GA between weeks 22–31 (both included) and late preterm birth as births between weeks 32–36 (both included). When information on GA was missing, we excluded the pregnancy. To evaluate the consequence of this exclusion, we performed a sensitivity analysis replacing missing GA with median GA. We defined miscarriage as having a diagnosis of miscarriage in the DNPR and a GA between 7 and 21 weeks (both included). We did not include miscarriages before week 7 because of incomplete registration of early abortions [20].

From the DMBR, we retrieved information on maternal age and smoking status. Infants born to smokers have lower median birth weight than those born to non-smokers [21] and smoking may be a risk factor for surgery [22]. Since smoking status was not available from the DNPR, we lacked smoking information for pregnancies resulting in miscarriages.

### Statistical analyses

We tabulated maternal characteristics and fetal vital status for pregnancies with appendectomy, cholecystectomy and diagnostic laparoscopy 0–11, 12–23 and 24+ months before estimated day of LMP and calculated absolute risk (AR) and risk difference (RD) of SGA, early preterm birth, late preterm birth and miscarriage for all groups. We used logistic regression analysis to calculate odds ratios (ORs) of the association of timing of surgery (appendectomy, cholecystectomy and diagnostic laparoscopy, respectively), with surgery > 24 months before LMP as reference, and risk of SGA, late preterm birth, early preterm birth, and miscarriage after week 7, respectively. In the regression analysis, we adjusted for maternal smoking and maternal age using multiple imputation to account for missing information on smoking status. We tabulated diagnosis codes related to diagnostic laparoscopy and performed a regression analysis for the two main diagnosis groups. We performed an analysis with only complete cases of smoking status and an analysis with women with appendectomies performed more than 5 years before pregnancy as reference group as sensitivity analyses. For miscarriages, we adjusted for maternal age. We stratified the analysis by year of surgery (1996–1999, 2000–2003, 2004–2007, 2008–2011, 2012–2015) for appendectomies, cholecystectomies and diagnostic laparoscopies conducted anytime between 0 and 23 months compared with the same surgery > 24 months before LMP.

We used the statistical software package STATA (version 13, Stata Corp., College Station, Texas, USA) for data analysis.

## Results

**Fig. 1:**
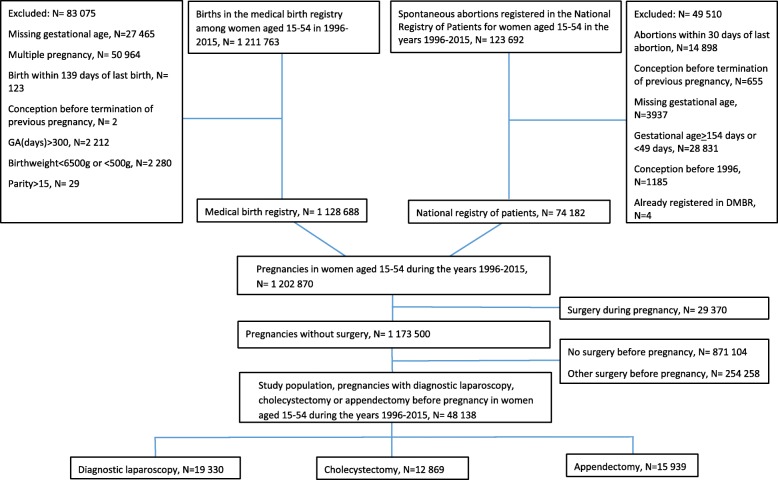
Inclusion of pregnancies

Among 1,173,500 pregnancies without major surgery during pregnancy from Denmark in 1996–2015, we identified 15,939 with appendectomy before LMP, 12869 with cholecystectomy before LMP and 19,330 with diagnostic laparoscopy before LMP (see Fig. 1). We excluded 2.4% of pregnancies in the main analysis due to missing information on GA.

Table 1 presents demographic characteristics of the study-population with appendectomy. Women with appendectomy more than 24 months before LMP were slightly older and more often multiparous than women with appendectomy performed less than 24 months before LMP (Table 1). Body-mass index was comparable in all groups, with a high prevalence of missing values (33.2–50.7%). The prevalence of smoking did not vary by timing of previous surgery, but the prevalence of inflammatory disease and diabetes was lowest in pregnancies with LMP more than 24 months after surgery. Smoking status was missing in 1707 (10.7%) of pregnancies with appendectomy prior to LMP. Demographic characteristics of the study-populations with cholecystectomy and diagnostic laparoscopy can be found in Additional Files 3 and 4, respectively.

**Table 1:** Demographic information on women with appendectomy before pregnancy

		Time in months from appendectomy to conception		
	Pregnancies with appendectomy before (%)	0–11	12–23	24+
Maternal characteristics	N (%)	N (%)	N (%)	N (%)
Total number of pregnancies	15,939 (100)	1694 (100)	1602 (100)	12,643 (100)
Maternal age, years				
< 20	231 (1.4)	88 (5.2)	73 (4.6)	70 (0.6)
20–29	8207 (51.5)	940 (55.5)	914 (57.1)	6353 (50.2)
30–39	7192 (45.1)	629 (37.1)	596 (37.2)	5967 (47.2)
40–49	309 (1.9)	37 (2.2)	19 (1.2)	253 (2.0)
Parity				
Nulliparous	6285 (39.4)	714 (42.1)	691 (43.1)	4880 (38.6)
Multiparous	8781 (55.1)	884 (52.2)	811 (50.6)	7086 (56.0)
Missing information on parity	873 (5.5)	96 (5.7)	100 (6.2)	677 (5.4)
BMI, kg/m2				
< 18.5	457 (2.9)	63 (3.7)	36 (2.2)	358 (2.8)
18.5–24.9	6099 (38.3)	483 (28.5)	480 (30.0)	5136 (40.6)
25–29.9	2166 (13.6)	195 (11.5)	177 (11.0)	1794 (14.2)
> =30	1363 (8.6)	115 (6.8)	96 (6.0)	1152 (9.1)
Missing information on BMI	5854 (36.7)	838 (49.5)	813 (50.7)	4203 (33.2)
Smoking status				
Non-smokers	11,506 (72.2)	1107 (65.3)	1050 (65.5)	9349 (73.9)
Smoking during pregnancy	2726 (17.1)	315 (18.6)	299 (18.7)	2112 (16.7)
Missing information on smoking status	1707 (10.7)	272 (16.1)	253 (15.8)	1182 (9.3)
Maternal disease				
Diabetes	64 (0.4)	13 (0.8)	9 (0.6)	42 (0.3)
Inflammatory disease	133 (0.8)	24 (1.4)	25 (1.6)	84 (0.7)
Vital status				
Liveborn	15,019 (94.2)	1593 (94.0)	1497 (93.4)	11,929 (94.4)
Stillborn	47 (0.3)	5 (0.3)	5 (0.3)	37 (0.3)
Missing information on vital status	873 (5.5)	96 (5.7)	100 (6.2)	677 (5.4)

**Fig. 2:**
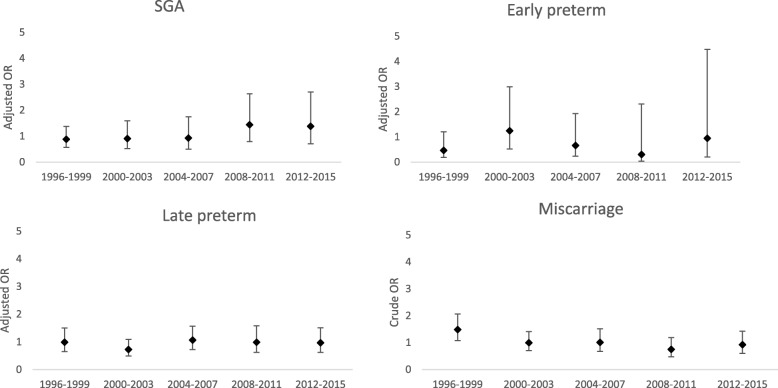
Development in odds ratio (OR) with 95% confidence interval (CI) by year-group of surgery. Legend: Adjusted odds ratio (aOR) of small for gestational age (SGA), early preterm birth, late preterm birth and crude odds ratio (cOR) of miscarriage among women who underwent appendectomy 0–23 months before pregnancy, compared with women who underwent appendectomy at least 24 months before pregnancy from 1996 to 2015

The OR of SGA, early preterm birth, late preterm birth and miscarriage in women with appendectomy less than 24 months before LMP compared with 24 months or more before LMP did not substantially change over calendar-time (see Fig. 2). The same figures for cholecystectomy and diagnostic laparoscopy, respectively, can be seen in Additional Files 5 and 6.

The risk of SGA was 2.2% when the procedure was conducted 0–11 months before LMP, 3.2% 12–23 months before LMP compared with 2.2% more than 24 months before LMP, corresponding to adjusted ORs of 0.9 (95% CI; 0.7 to 1.3) and 1.4 (95% CI;1.0 to 1.9), respectively. For late preterm birth the risks were 4.8, 4.4 and 4.7%, respectively and for early preterm birth 0.7, 0.5, and 0.8%, respectively. The adjusted ORs for late preterm and early preterm birth varied between 0.6 and 1.0. The risks for miscarriages after GA week 7 were 5.7, 6.2 and 5.4% with adjusted ORs of 1.1(95% CI;0.9 to 1.4) and 1.3(95% CI;1.0 to 1.6), respectively (see Table 2). Replacing missing GA with the median GA, yielded comparable results as did complete-case analysis regarding smoking status. Changing the reference group to women with appendectomies performed more than 5 years before pregnancy yielded similar results (aORs of SGA 0.9 (95% CI; 0.6 to 1.3), early preterm birth 1.0 (95% CI; 0.5 to 1.9), late preterm birth 1.0 (95% CI; 0.8 to 1.3) and miscarriage 1.2 (95% CI, 0.9 to 1.5).

**Table 2:** Odds ratios of adverse birth outcomes after appendectomy before pregnancy

Outcome	Months from appendectomy to LMP			Appendectomy 0–11 months before LMP		Appendectomy 12–23 months before LMP	
	0–11	12–23	24+				
	N (%)	N (%)	N (%)	cOR (95% CI)	aOR^*^ (95% CI)	cOR (95% CI)	aOR^*^ (95% CI)
SGA	37/1694 (2.2)	51/1602 (3.2)	279/12643 (2.2)	0.99 (0.70;1.41)	0.92 (0.65;1.31)	1.47 (1.09;1.99)	1.37 (1.00;1.86)
Preterm							
Early preterm	12/1694 (0.7)	8/1602 (0.5)	97/12643 (0.8)	0.92 (0.51;1.68)	0.88 (0.48;1.61)	0.65 (0.32;1.34)	0.61 (0.29;1.26)
Late preterm	82/1694 (4.8)	70/1602 (4.4)	599/12643 (4.7)	1.02 (0.81;1.30)	0.98 (0.77;1.24)	0.92 (0.71;1.18)	0.86 (0.67;1.11)
Miscarriage	96/1694 (5.7)	100/1602 (6.2)	677/12643 (5.4)	1.06 (0.85;1.32)	1.13 (0.90;1.40)	1.18 (0.95;1.46)	1.27 (1.02;1.58)

^*^Adjusted for smoking status and maternal age, miscarriage only adjusted for maternal age

Legend: Prevalence, crude and adjusted odds ratios (cOR/aOR) of small for gestational age (SGA), early preterm birth, late preterm birth and miscarriage in pregnancies with appendectomy before pregnancy

Timing of cholecystectomy before LMP did not affect the risk of adverse birth outcomes markedly. We found an aOR of miscarriage in pregnancies 0–11 months after cholecystectomy on 1.3 (95% CI;1.1 to 1.5) and early preterm birth in pregnancies 12–23 months after cholecystectomy on 1.2 (95% CI; 0.8 to 2.0)) (Table 3). For diagnostic laparoscopy we found increased risk of SGA (risk difference (RD) 1.5%, adjusted OR 1.6 (95% CI;1.3 to 1.9)) when pregnancy was 0–11 month after the procedure (Table 4) and for early preterm birth (RD 0.6%, 1.6 (95% CI;1.1 to 2.2)) when pregnancy was 12–23 months after the procedure (Table 4). The most common diagnoses related to diagnostic laparoscopy were urinary-tract and genital disorders (45.0%) and unspecified symptoms and findings (27.3%) (see Additional File 7). The risk of SGA, early preterm birth, late preterm birth and miscarriage is evaluated for these two main diagnosis groups in diagnostic laparoscopies in Additional file 8.

**Table 3:** Odds ratios of adverse birth outcomes after cholecystectomy before pregnancy

Outcome	Months from cholecystectomy to LMP			Cholecystectomy 0–11 months before LMP		Cholecystectomy 12–23 months before LMP	
	0–11	12–23	24+				
	N (%)	N (%)	N (%)	cOR (95% CI)	aOR^*^ (95% CI)	cOR (95% CI)	aOR^*^ (95% CI)
SGA	75/2950 (2.5)	47/2243 (2.1)	181/7676 (2.4)	1.1 (0.8;1.4)	1.1 (0.8;1.5)	0.9 (0.6;1.2)	0.9 (0.6;1.2)
Preterm							
Early preterm	29/2950 (1.0)	24/2243 (1.1)	68/7676 (0.9)	1.1 (0.7;1.7)	1.1 (0.7;1.8)	1.2 (0.8;1.9)	1.2 (0.8;2.0)
Late preterm	108/2950 (3.7)	103/2243 (4.6)	364/7676 (4.7)	0.8 (0.6;1.0)	0.8 (0.6;1.0)	1.0 (0.8;1.2)	1.0 (0.8;1.2)
Miscarriage	227/2950 (7.7)	136/2243 (6.1)	526/7676 (6.9)	1.1 (1.0;1.3)	1.3 (1.1;1.5)	0.9 (0.7;1.1)	1.0 (0.8;1.2)

^*^Adjusted for smoking status and maternal age, miscarriage only adjusted for maternal age

Legend: Prevalence, crude and adjusted odds ratios (cOR/aOR) of small for gestational age (SGA), early preterm birth, late preterm birth and miscarriage in pregnancies with cholecystectomy before pregnancy

**Table 4:** Odds ratios of adverse birth outcomes after diagnostic laparoscopy before pregnancy

Outcome	Months from diagnostic laparoscopy to LMP			Diagnostic laparoscopy 0–11 months before LMP		Diagnostic laparoscopy 12–23 months before LMP	
	0–11	12–23	24+				
	N (%)	N (%)	N (%)	cOR (95% CI)	aOR^*^ (95% CI)	cOR (95% CI)	aOR^*^ (95% CI)
SGA	166/4199 (4.0)	81/2848 (2.8)	313/12283 (2.5)	1.6 (1.3;1.9)	1.6 (1.3;1.9)	1.1 (0.9;1.4)	1.1 (0.9;1.4)
Preterm							
Early preterm	50/4199 (1.2)	42/2848 (1.5)	112/12283 (0.9)	1.3 (0.9;1.8)	1.2 (0.9;1.7)	1.6 (1.1;2.3)	1.6 (1.1;2.2)
Late preterm	249/4199 (5.9)	142/2848 (5.0)	593/12283 (4.8)	1.2 (1.1;1.4)	1.2 (1.0;1.4)	1.0 (0.9;1.2)	1.0 (0.8;1.2)
Miscarriage	281/4199 (6.7)	209/2848 (7.3)	875/12283 (7.1)	0.9 (0.8;1.1)	1.0 (0.9;1.2)	1.0 (0.9;1.2)	1.1 (1.0;1.3)

^*^Adjusted for smoking status and maternal age, miscarriage only adjusted for maternal age

Legend: Prevalence, crude and adjusted odds ratios (cOR/aOR) of small for gestational age (SGA), early preterm birth, late preterm birth and miscarriage in pregnancies with diagnostic laparoscopy before pregnancy

## Discussion

In this population-based study with more than 46,000 pregnancies with surgery before LMP, we found no major elevated risks of SGA, early preterm birth, late preterm birth and miscarriage after GA week 7 following appendectomy and cholecystectomy. For diagnostic laparoscopy, an association with SGA, early preterm birth and late preterm birth, could not be excluded. However, the precision of our estimates do not allow us to draw any firm conclusions.

Disentangling the effect of surgery from the effect of the underlying condition is difficult and causal mechanisms are uncertain. However, if the laparoscopic procedure itself is associated with elevated risks, we would expect increased risks following appendectomy and cholecystectomy as well. The possibly increased risks following diagnostic laparoscopy could therefore likely be explained by confounding by underlying indications. We did not analyze open and laparoscopic procedures separately due to small numbers, but Ibiebele et al. [2] showed that the risk of SGA was comparable in laparoscopy and laparotomy. Hence, it seems applicable to investigate adverse birth outcomes of these two types of surgery in one category.

To our knowledge, no previous studies have addressed these questions, and our findings thus adds to the existing literature. A cohort study showed an association between previous bariatric surgery and adverse obstetric outcomes [8], but indication and effects of bariatric surgery are different, and are thus not directly comparable.

It is a strength of our study that the risk of selection bias is low as the included registries are virtually complete [10, 13]. However, we did not include abortions before week 7 because they are likely to be underreported [20]. This makes us unable to investigate the risk of abortion between LMP and week 7 of gestation. Information on GA is missing in 2.4% of all the included pregnancies. We used this variable for calculation of both SGA and the time of exposure before pregnancy and chose complete-case analysis for our main results. An analysis with replacement of missing GA with the median value of GA, yielded comparable results. We used multiple imputation [23, 24] of missing data on the potential confounder smoking (10.7%). Complete-case analysis did not lead to substantially different estimates; we therefore find it unlikely that residual confounding by smoking can explain our lack of an association.

It is a limitation to our study that we lacked information on maternal complications during or after surgery, since such complications could affect the risk of adverse birth outcomes in following pregnancies. However, as we examined the risk of adverse birth outcomes after the last surgery before pregnancy, there would be no surgical complications to these. The fact, that diagnostic laparoscopy is conducted on a variety of indications which may to varying degree be present during a following pregnancy prevents a firm conclusion regarding the actual impact of surgery itself on adverse birth outcomes. Additional to varying indications for surgery, different types of anesthesia might also influence birth outcomes in subsequent pregnancies [25].

More hypotheses can explain why the post-surgical changes in maternal microbiome and pro-inflammatory mediators known in relation to surgery do not seem to increase the risk of adverse obstetrical outcomes [7, 26] when appendectomy or cholecystectomy is conducted before pregnancy. The changes may be so fast reversible, that no effect is seen even in pregnancy immediately after surgery [27]. The changes might also induce an altered fetal microbiome that could even increase the fetal weight [28], or the occurring alterations are simply too small to affect the outcome measures significantly. Dietary and pregnancy-induced changes in the microbiome [29, 30] could also by far exceed the changes induced by surgery.

## Conclusions

Based on our findings, we conclude, that it appears safe to conceive any time after appendectomy and cholecystectomy regarding obstetric outcomes. Probably depending on indication, attention should be payed 0–11 months after diagnostic laparoscopy.

## Additional Files

**Additional file 1.** Surgical codes used to identify minor procedures and procedures related to birth and fetal diagnostics. These procedures were not included in the definition of surgery during pregnancy.

**Additional file 2.** Surgical codes used to identify, appendectomies, cholecystectomies, and diagnostic laparoscopy, respectively.

**Additional file 3.** Demographic information on women with cholecystectomy before pregnancy

**Additional file 4.** Demographic information on women with diagnostic laparoscopy before pregnancy

**Additional file 5.** Development in odds ratio (OR) with 95% confidence interval (CI) by year-group of surgery. Legend: Adjusted odds ratio (aOR) of small for gestational age (SGA), early preterm birth, late preterm birth and miscarriage among women who underwent cholecystectomy 0–23 months before pregnancy, compared with women who underwent cholecystectomy at least 24 months before pregnancy from 1996 to 2015

**Additional file 6.** Development in odds ratio (OR) with 95% confidence interval (CI) by year-group of surgery. Legend: Adjusted odds ratio (aOR) of small for gestational age (SGA), early preterm birth, late preterm birth and miscarriage among women who underwent diagnostic laparoscopy 0–23 months before pregnancy, compared with women who underwent diagnostic laparoscopy at least 24 months before pregnancy from 1996 to 2015

**Additional file 7.** Main diagnosis groups in women with diagnostic laparoscopy from 1996 to 2015. Legend: The diagram shows the prevalence (%) of diagnosis groups among diagnostic laparoscopies from 1996 onwards in total, diagnostic laparoscopies conducted 0–11 months before pregnancy, 12–23 months before pregnancy and more than 24 months before pregnancy.

**Additional file 8.** Odds ratios of adverse birth outcomes after diagnostic laparoscopy before pregnancy when diagnosis was urogenital disorder or unspecific symptoms. Legend: Prevalence, crude and adjusted odds ratios (cOR/aOR) of small for gestational age (SGA), early preterm birth, late preterm birth and miscarriage in pregnancies with diagnostic laparoscopy before pregnancy when diagnosis was urogenital disorder or unspecific symptoms

## Data Availability

The data that support the findings of this study are available from the Danish Health Data Board (Sundhedsdatastyrelsen) but restrictions apply to the availability of these data, which were used under license for the current study, and so are not publicly available.

## Abbreviations

AR: Absolute risk
DMBR: Danish Medical Birth Registry
DNPR: Danish National Patient Registry
GA: Gestational age
ICD-10: International Classification of Diseases 10th revision
ICD-8: International Classification of Diseases 8th revision
LMP: Last menstrual period
OR: Odds ratio
RD: Risk difference
SGA: Small for gestational age
