# Supplementary material for: Appendectomy, cholecystectomy and diagnostic laparoscopy conducted before pregnancy and risk of adverse birth outcomes: a nationwide registry-based prevalence study 1996–2015
Source: BMC Pregnancy Childbirth. 2020 Feb 13;20:108. doi: 10.1186/s12884-020-2796-3 (PMC7020513; doi:10.1186/s12884-020-2796-3)
Supplement: Supplementary file 1 — Additional file 1. Surgical codes used to identify minor procedures and procedures related to birth and fetal diagnostics. These procedures were not included in the definition of surgery during pregnancy. [file 12884_2020_2796_MOESM1_ESM.pdf]

Additional file 1

Surgical codes used to identify minor procedures and procedures related to birth and fetal diagnostics. These procedures were not included in the definition of surgery during pregnancy.

**Type of surgery**	**Name of procedure**	**Surgical code (NOMESCO)**
Minor non-obstetric surgery	Surgery on peripheral nerves	KACA, KACB, KACC, KACW
	Biopsy of thyroid gland	KBAA00
	Eye and adjacent structures	KC
	Selected surgeries on ear, nose and larynx	KDA, KDB, KDHA, KDHB00, KDHB05, KDHB10, KDHB20, KDHC, KDHD10, KDJA10, KDJD30
	Selected surgeries on teeth, jaws, mouth and pharynx	KEA, KEB, KEC, KEDC32, KEDC34, KEDC35A, KEDC35B, KEGC35B, KEHA, KEJA, KEKA, KELA
	Incision, suture and biopsy of mammary gland	KHAA
	Biopsies on digestive system	KJJA20, KJLA10, KJMB00
	Selected surgeries on urinary system, male genital organs and retroperitoneal space	KKAB, KKAT, KKBB, KKBT, KKCB, KKCT, KKE, KKF, KKG, KKKA10
	Puncture of ovary, infertility operations, operations for tubal pregnancy, cervix reconstruction, biopsy of uterus and uterine ligaments, termination of pregnancy, dilatation, curettage and biopsy of cervix uteri, excision or destruction of lesion of cervix	KLAA00, KLAA03, KLAA06, KLAA10, KLAA11, KLAA20, KLAA96, KLBF, KLBC, KLDD10A, KLDD10B, KLCA10, KLCA13, KLCA16, KLCA30, KLCA31, KLCH, KLDA, KLDB, KLEB00, KLFB

	uteri, biopsy of vagina, biopsy, excision or destruction of lesion of vulva and perineum<br><br>Surgery on peripheral vessels and lymphatic system<br>Minor surgical procedures<br>Skin<br>Transluminal endoscopy	KPCT, KPDT, KPHT<br><br>KT<br>KQ<br>KU
Procedures related to birth and fetal diagnostics	Amniocentesis, chorion villus biopsy, external version, surgical induction or advancement of labour, vacuum delivery, forceps cephalic delivery, breech delivery, various procedure relating to delivery, procedures after delivery or abortion, cesarean section and obstetric laparotomies, re-evacuatio after birth or spontaneous abortion	KMAA00, KMAA10, KMAB10, KMAB20, KMAC, KMAD, KMAE, KMAF, KMAG, KMAH, KMB, KMC, KMWW00

Pregnancies with any of the above mentioned surgical codes (coded according to the Nordic Medico-Statistical Committee (NOMESCO) classification) are considered unexposed to surgery.
