# Supplementary material for: Appendectomy, cholecystectomy and diagnostic laparoscopy conducted before pregnancy and risk of adverse birth outcomes: a nationwide registry-based prevalence study 1996–2015
Source: BMC Pregnancy Childbirth. 2020 Feb 13;20:108. doi: 10.1186/s12884-020-2796-3 (PMC7020513; doi:10.1186/s12884-020-2796-3)
Supplement: Supplementary file 2 — Additional file 2. Surgical codes used to identify, appendectomies, cholecystectomies, and diagnostic laparoscopy, respectively. [file 12884_2020_2796_MOESM2_ESM.pdf]

Additional file 2

Surgical codes used to identify, appendectomies, cholecystectomies, and diagnostic laparoscopy, respectively.

Type of surgery	Surgical code (NOMESCO or Operations and treatment Classification)	
Appendectomy	KJEA<br>43000<br>43001	Appendectomy<br>Appendectomia<br>Appendectomia endoscopica
Cholecystectomy	KJKA20<br>KJKA21<br>47360<br>47365<br>53500	Cholecystectomy<br>Laparoscopic cholecystectomy<br>Cholecystectomia<br>Cholecystectomia endoscopica<br>Cholecystectomia
Diagnostic laparoscopy	KJAH01<br>40240<br>40420<br>93190	Laparoscopy<br>Laparoscopia<br>Laparoscopia<br>Laparoscopia

Surgeries were coded according to the Nordic Medico-Statistical Committee (NOMESCO) classification from 1996 and onwards and according to the Operations and treatment Classification before 1996.
