# Supplementary material for: Appendectomy, cholecystectomy and diagnostic laparoscopy conducted before pregnancy and risk of adverse birth outcomes: a nationwide registry-based prevalence study 1996–2015
Source: BMC Pregnancy Childbirth. 2020 Feb 13;20:108. doi: 10.1186/s12884-020-2796-3 (PMC7020513; doi:10.1186/s12884-020-2796-3)
Supplement: Supplementary file 3 — Additional file 3. Demographic information on women with cholecystectomy before pregnancy [file 12884_2020_2796_MOESM3_ESM.docx]

|  |  |  | Time in months from cholecystectomy to pregnancy | | | | | |
| --- | --- | --- | --- | --- | --- | --- | --- | --- |
|  | **Pregnancies with cholecystectomy before (%)** | | 0-11 | | 12-23 | | 24+ | |
| Maternal characteristics | | |  |  |  |  |  |  |
| Total number of pregnancies | 12869(100) | | 2950(100) | | 2243(100) | | 7676(100) | |
| Maternal age, years | |  |  |  |  |  |  |  |
| <20 | 41(0.3) | | 19(0.6) | | 11(0.5) | | 11(0.1) | |
| 20-29 | 4812(37.4) | | 1410(47.8) | | 1007(44.9) | | 2395(31.2) | |
| 30-39 | 7451(57.9) | | 1446(49.0) | | 1164(51.9) | | 4841(63.1) | |
| 40-49 | 563(4.4) | | 74(2.5) | | 61(2.7) | | 428(5.6) | |
| >=50 | <5(0.0) | | <5(0.0) | | <5(0.0) | | <5(0.0) | |
| Parity |  | |  | |  | |  | |
| Nulliparity | 2931(22.8) | | 614(20.8) | | 500(22.3) | | 1817(23.7) | |
| Multiparity | 9039(70.2) | | 2106(71.4) | | 1605(71.6) | | 5328(69.4) | |
| Missing information on parity | 899(7.0) | | 230(7.8) | | 138(6.2) | | 531(6.9) | |
| BMI, kg/m2 | |  |  |  |  |  |  |  |
| <18.5 | 137(1.1) | | 26(0.9) | | 26(1.2) | | 85(1.1) | |
| 18.5-24.9 | 2740(21.3) | | 515(17.5) | | 410(18.3) | | 1815(23.6) | |
| 25-29.9 | 2364(18.4) | | 492(16.7) | | 398(17.7) | | 1474(19.2) | |
| >=30 | 3309(25.7) | | 654(22.2) | | 533(23.8) | | 2122(27.6) | |
| Missing information on BMI | 4319(33.6) | | 1263(42.8) | | 876(39.1) | | 2180(28.4) | |
| Smoking status |  | |  | |  | |  | |
| Non-smokers | 8764(68.1) | | 1944(65.9) | | 1512(67.4) | | 5308(69.2) | |
| Smoking during pregnancy | 2631(20.4) | | 570(19.3) | | 465(20.7) | | 1596(20.8) | |
| Missing information on smoking status | 1474(11.5) | | 436(14.8) | | 266(11.9) | | 772(10.1) | |
| Maternal disease |  | |  | |  | |  | |
| Diabetes | 234(1.8) | | 77(2.6) | | 48(2.1) | | 109(1.4) | |
| Inflammatory disease | 131(1.0) | | 30(1.0) | | 32(1.4) | | 69(0.9) | |
| Vital status |  | |  | |  | |  | |
| Liveborn | 11920(92.6) | | 2712(91.9) | | 2096(93.4) | | 7112(92.7) | |
| Stillborn | 50(0.4) | | 8(0.3) | | 9(0.4) | | 33(0.4) | |
| Missing information on vital status | 899(7.0) | | 230(7.8) | | 138(6.2) | | 531(6.9) | |
