# Supplementary material for: Appendectomy, cholecystectomy and diagnostic laparoscopy conducted before pregnancy and risk of adverse birth outcomes: a nationwide registry-based prevalence study 1996–2015
Source: BMC Pregnancy Childbirth. 2020 Feb 13;20:108. doi: 10.1186/s12884-020-2796-3 (PMC7020513; doi:10.1186/s12884-020-2796-3)
Supplement: Supplementary file 4 — Additional file 4. Demographic information on women with diagnostic laparoscopy before pregnancy [file 12884_2020_2796_MOESM4_ESM.docx]

|  |  |  | Time in months from diagnostic laparoscopy to conception | | | | | |
| --- | --- | --- | --- | --- | --- | --- | --- | --- |
|  | **Pregnancies with diagnostic laparoscopy before (%)** | | 0-11 | | 12-23 | | 24+ | |
| Maternal characteristics | | |  |  |  |  |  |  |
| Total number of pregnancies | 19330(100) | | 4199(100) | | 2848(100) | | 12283(100) | |
| Maternal age, years | |  |  |  |  |  |  |  |
| <20 | 171(0.9) | | 81(1.9) | | 52(1.8) | | 38(0.3) | |
| 20-29 | 7471(38.6) | | 1952(46.5) | | 1308(45.9) | | 4211(34.3) | |
| 30-39 | 10952(56.7) | | 2062(49.1) | | 1417(49.8) | | 7473(60.8) | |
| 40-49 | 733(3.8) | | 104(2.5) | | 71(2.5) | | 558(4.5) | |
| >=50 | <5(0.0) | | <5(0.0) | | <5(0.0) | | 3(0.0) | |
| Parity |  | |  | |  | |  | |
| Nulliparity | 7199(37.2) | | 2172(51.7) | | 1333(46.8) | | 3694(30.1) | |
| Multiparity | 10747(55.6) | | 1741(41.5) | | 1301(45.7) | | 7705(62.7) | |
| Missing information on parity | 1384(7.2) | | 286(6.8) | | 214(7.5) | | 884(7.2) | |
| BMI, kg/m2 | |  |  |  |  |  |  |  |
| <18.5 | 436(2.3) | | 115(2.7) | | 57(2.0) | | 264(2.1) | |
| 18.5-24.9 | 5523(28.6) | | 1121(26.7) | | 713(25.0) | | 3689(30.0) | |
| 25-29.9 | 2226(11.5) | | 447(10.6) | | 286(10.0) | | 1493(12.2) | |
| >=30 | 1379(7.1) | | 243(5.8) | | 180(6.3) | | 956(7.8) | |
| Missing information on BMI | 9766(50.5) | | 2273(54.1) | | 1612(56.6) | | 5881(47.9) | |
| Smoking status |  | |  | |  | |  | |
| Non-smokers | 12316(63.7) | | 2643(62.9) | | 1650(57.9) | | 8023(65.3) | |
| Smoking during pregnancy | 3815(19.7) | | 825(19.6) | | 559(19.6) | | 2431(19.8) | |
| Missing information on smoking status | 3199(16.5) | | 731(17.4) | | 639(22.4) | | 1829(14.9) | |
| Maternal disease |  | |  | |  | |  | |
| Diabetes | 104(0.5) | | 33(0.8) | | 24(0.8) | | 47(0.4) | |
| Inflammatory disease | 124(0.6) | | 23(0.5) | | 17(0.6) | | 84(0.7) | |
| Vital status |  | |  | |  | |  | |
| Liveborn | 17879(92.5) | | 3898(92.8) | | 2618(91.9) | | 11363(92.5) | |
| Stillborn | 67(0.3) | | 15(0.4) | | 16(0.6) | | 36(0.3) | |
| Missing information on vital status | 1384(7.2) | | 286(6.8) | | 214(7.5) | | 884(7.2) | |
