# Supplementary material for: Appendectomy, cholecystectomy and diagnostic laparoscopy conducted before pregnancy and risk of adverse birth outcomes: a nationwide registry-based prevalence study 1996–2015
Source: BMC Pregnancy Childbirth. 2020 Feb 13;20:108. doi: 10.1186/s12884-020-2796-3 (PMC7020513; doi:10.1186/s12884-020-2796-3)

Adjusted odds ratios (ORs) of small for gestational age (SGA), early preterm and late preterm birth and crude OR of miscarriage by year of surgery in pregnancies with diagnostic laparoscopy 0-23 months before pregnancy compared with pregnancies with diagnostic laparoscopy more than 24 months before pregnancy

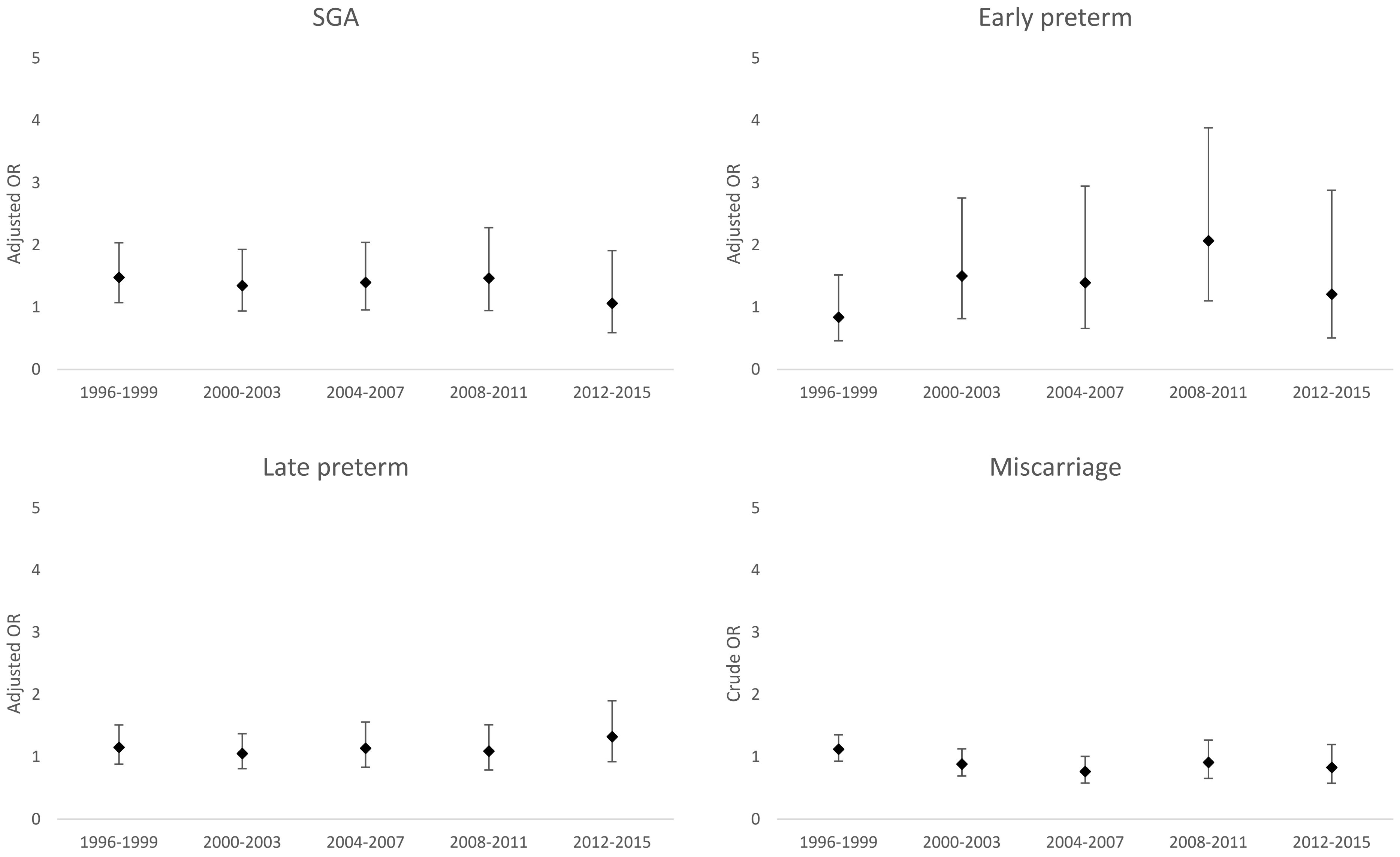

Supplement: Supplementary file 6 — Additional file 6. Development in odds ratio (OR) with 95% confidence interval (CI) by year-group of surgery. Legend: Adjusted odds ratio (aOR) of small for gestational age (SGA), early preterm birth, late preterm birth and miscarriage among women who underwent diagnostic laparoscopy 0–23 months before pregnancy, compared with women who underwent diagnostic laparoscopy at least 24 months before pregnancy from 1996 to 2015 [file 12884_2020_2796_MOESM6_ESM.pdf]
