# Supplementary material for: Appendectomy, cholecystectomy and diagnostic laparoscopy conducted before pregnancy and risk of adverse birth outcomes: a nationwide registry-based prevalence study 1996–2015
Source: BMC Pregnancy Childbirth. 2020 Feb 13;20:108. doi: 10.1186/s12884-020-2796-3 (PMC7020513; doi:10.1186/s12884-020-2796-3)
Supplement: Supplementary file 7 — Additional file 7. Main diagnosis groups in women with diagnostic laparoscopy from 1996 to 2015. Legend: The diagram shows the prevalence (%) of diagnosis groups among diagnostic laparoscopies from 1996 onwards in total, diagnostic laparoscopies conducted 0–11 months before pregnancy, 12–23 months before pregnancy and more than 24 months before pregnancy. [file 12884_2020_2796_MOESM7_ESM.pdf]

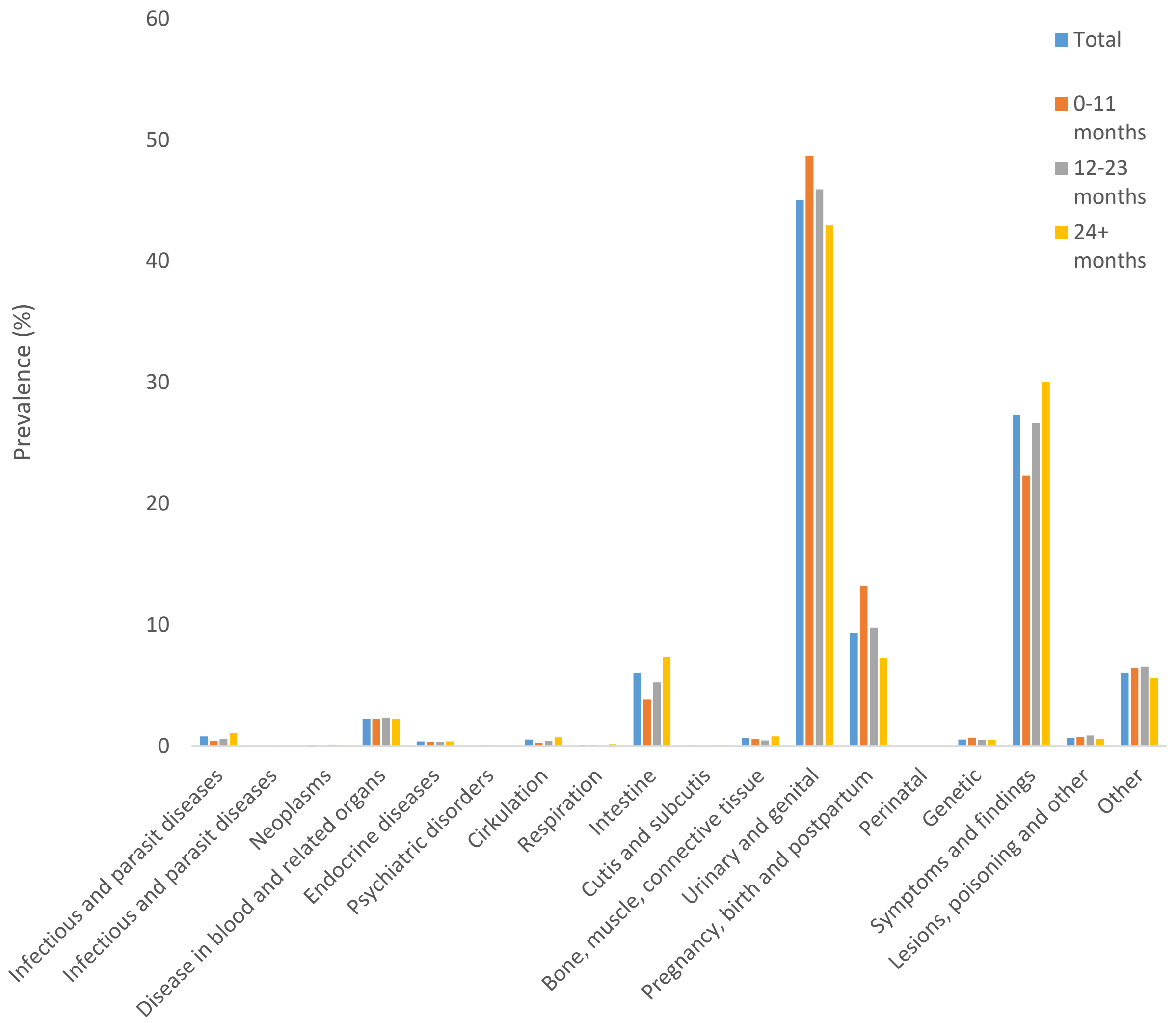

Legend: The diagram shows the prevalence (%) of diagnosis groups among diagnostic laparoscopies from 1996 onwards in total, diagnostic laparoscopies conducted 0-11 months before pregnancy, 12-23 months before pregnancy and more than 24 months before pregnancy.
