# Supplementary material for: Appendectomy, cholecystectomy and diagnostic laparoscopy conducted before pregnancy and risk of adverse birth outcomes: a nationwide registry-based prevalence study 1996–2015
Source: BMC Pregnancy Childbirth. 2020 Feb 13;20:108. doi: 10.1186/s12884-020-2796-3 (PMC7020513; doi:10.1186/s12884-020-2796-3)
Supplement: Supplementary file 8 — Additional file 8. Odds ratios of adverse birth outcomes after diagnostic laparoscopy before pregnancy when diagnosis was urogenital disorder or unspecific symptoms. Legend: Prevalence, crude and adjusted odds ratios (cOR/aOR) of small for gestational age (SGA), early preterm birth, late preterm birth and miscarriage in pregnancies with diagnostic laparoscopy before pregnancy when diagnosis was urogenital disorder or unspecific symptoms [file 12884_2020_2796_MOESM8_ESM.docx]

Additional file 8. Odds ratios of adverse birth outcomes after diagnostic laparoscopy before pregnancy when diagnosis was urogenital disorder

| Outcome | Months from diagnostic laparoscopy to pregnancy | | | | Diagnostic laparoscopy 0-11 months before pregnancy | | Diagnostic laparoscopy 12-23 months before pregnancy | |
| --- | --- | --- | --- | --- | --- | --- | --- | --- |
|  | **0-11** | **12-23** | **24+** |  | |  |  |  |
|  | N (%) | N (%) | N (%) | cOR (95% CI) | | aOR^*^ (95% CI) | cOR (95% CI) | aOR^*^ (95% CI) |
| SGA | 99/2119(4.7) | 52/1543(3.4) | 200/7680(2.6) | 1.8(1.4;2.3) | | 1.8(1.4;2.4) | 1.3(1.0;1.8) | 1.3(1.0;1.8) |
| Preterm | | | | | | | | |
| Early preterm | 19/2119 (0.9) | 25/1543(1.6) | 67/7680(0.9) | 1.0(0.6;1.7) | | 1.0(0.6;1.6) | 1.9(1.2;3.0) | 1.8(1.1;2.9) |
| Late preterm | 125/2119 (5.9) | 69/1543(4.5) | 349/7680(4.5) | 1.3(1.1;1.6) | | 1.3(1.0;1.6) | 1.0(0.8;1.3) | 1.0(0.7;1.3) |
| Miscarriage | 145/2119 (6.8) | 133/1543(8.6) | 605/7680(7.9) | 0.9(0.7;1.0) | | 0.9(0.8;1.1) | 1.1(0.9;1.3) | 1.2(1.0;1.5) |

^*^Adjusted for smoking status and maternal age, miscarriage only adjusted for maternal age

Odds ratios of adverse birth outcomes after diagnostic laparoscopy before pregnancy when diagnosis was unspecific symptoms

| Outcome | Months from diagnostic laparoscopy to pregnancy | | | | Diagnostic laparoscopy 0-11 months before pregnancy | | Diagnostic laparoscopy 12-23 months before pregnancy | |
| --- | --- | --- | --- | --- | --- | --- | --- | --- |
|  | **0-11** | **12-23** | **24+** |  | |  |  |  |
|  | N (%) | N (%) | N (%) | cOR (95% CI) | | aOR^*^ (95% CI) | cOR (95% CI) | aOR^*^ (95% CI) |
| SGA | 39/1051(3.7) | 32/1078(3.0) | 168/6641(2.5) | 1.5(1.0;2.1) | | 1.5(1.0;2.2) | 1.2(0.8;1.8) | 1.2(0.8;1.8) |
| Preterm | | | | | | | | |
| Early preterm | 13/1051 (1.2) | 13/1078 (1.2) | 69/6641 (1.0) | 1.2(0.7;2.2) | | 1.1(0.6;2.0) | 1.2(0.6;2.1) | 1.0(0.6;1.9) |
| Late preterm | 59/1051 (5.6) | 57/1078 (5.3) | 327/6641 (4.9) | 1.1(0.9;1.5) | | 1.1(0.8;1.5) | 1.1(0.8;1.4) | 1.0(0.8;1.4) |
| Miscarriage | 87/1051 (8.3) | 93/1078 (8.6) | 527/6641 (7.9) | 1.0(0.8;1.3) | | 1.3(2.8;5.4) | 1.1(0.9;1.4) | 1.3(1.0;1.6) |

^*^Adjusted for smoking status and maternal age, miscarriage only adjusted for maternal age

Abbreviations: CI, confidence interval; SGA, small for gestational age

Legend: Prevalence, crude and adjusted odds ratios (cOR/aOR) of small for gestational age (SGA), early preterm birth, late preterm birth and miscarriage in pregnancies with diagnostic laparoscopy before pregnancy when diagnosis was urogenital disorder or unspecific symptoms
